# Dichlorido(2,6-dipyrazol-1-ylpyridine)zinc(II)

**DOI:** 10.1107/S1600536808031152

**Published:** 2008-10-09

**Authors:** Zhong Nian Yang, Ting Ting Sun

**Affiliations:** a Department of Chemistry and Chemical Engineering, Binzhou University, Binzhou 256603, People’s Republic of China; b Department of Chemistry, Shandong Normal University, Jinan 250014, People’s Republic of China

## Abstract

In the title complex, [ZnCl_2_(C_11_H_9_N_5_)], the Zn^II^ ion assumes a distorted trigonal–bipyramidal ZnN_3_Cl_2_ coordination geometry [Zn—N = 2.1397 (16)–2.2117 (17) Å, Zn—Cl = 2.2470 (6) and 2.2564 (6) Å]. The crystal packing exhibits π–π stacking inter­actions between the 2,6-dipyrazol-1-ylpyridine ligands of neighbouring mol­ecules.

## Related literature

For the related crystal structure of dichlorido­[2,6-bis(pyrazol­yl­meth­yl)pyridine]zinc(II), see Balamurugan *et al.* (2004 ▶).
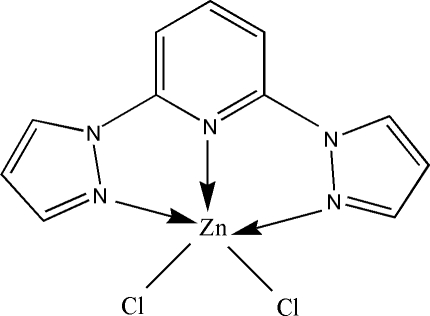


## Experimental

### 

#### Crystal data

[ZnCl_2_(C_11_H_9_N_5_)]
*M* *_r_* = 347.50
Monoclinic, 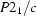

*a* = 10.9630 (17) Å
*b* = 8.0263 (13) Å
*c* = 14.943 (2) Å
β = 93.079 (2)°
*V* = 1313.0 (4) Å^3^
*Z* = 4
Mo *K*α radiation
μ = 2.27 mm^−1^
*T* = 298 (2) K
0.48 × 0.42 × 0.29 mm

#### Data collection

Bruker SMART APEX CCD diffractometer
Absorption correction: multi-scan (*SADABS*; Sheldrick, 1996 ▶) *T*_min_ = 0.409, *T*_max_ = 0.559 (expected range = 0.379–0.518)
7375 measured reflections
2848 independent reflections
2431 reflections with *I* > 2σ(*I*)
*R*_int_ = 0.030

#### Refinement

*R*[*F* ^2^ > 2σ(*F* ^2^)] = 0.029
*wR*(*F* ^2^) = 0.078
*S* = 1.05
2848 reflections
173 parameters
H-atom parameters constrained
Δρ_max_ = 0.28 e Å^−3^
Δρ_min_ = −0.32 e Å^−3^

### 

Data collection: *SMART* (Bruker, 1997 ▶); cell refinement: *SAINT* (Bruker, 1997 ▶); data reduction: *SAINT*; program(s) used to solve structure: *SHELXTL* (Sheldrick, 2008 ▶); program(s) used to refine structure: *SHELXTL*; molecular graphics: *SHELXTL*; software used to prepare material for publication: *SHELXTL* and local programs.

## Supplementary Material

Crystal structure: contains datablocks I, global. DOI: 10.1107/S1600536808031152/cv2454sup1.cif

Structure factors: contains datablocks I. DOI: 10.1107/S1600536808031152/cv2454Isup2.hkl

Additional supplementary materials:  crystallographic information; 3D view; checkCIF report

## Figures and Tables

**Table 1:** Selected interatomic distances (Å) *Cg*1, *Cg*2 and *Cg*3 are the centroids of the C4/N1/N4/N5/Zn1, C1–C3/N4/N5 and C4–C8/N1 rings, respectively.

*Cg*1⋯*Cg*2^i^	3.4087 (12)
*Cg*2⋯*Cg*3^i^	3.6253 (13)

Symmetry code: (i) 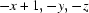.

**Fig. 1.**
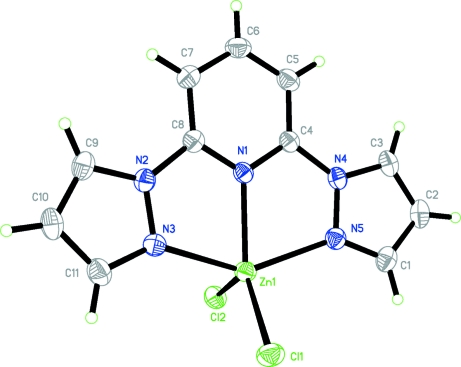
The molecular structure of (I) showing the atomic numbering and 30% probability displacement ellipsoids.

## supplementary crystallographic information

### Comment

2,6-Dipyrazol-1-ylpyridine and the relevant homologues as a tridentate ligand play an important role in modern coordination chemistry (Balamurugan *et al.*, 2004), and the interest in complexes with 2,6-dipyrazol-1-ylpyridine ligand stimulted us to prepare the title complex, (I). Herein we report its crystal structure.

In (I) (Fig. 1), each Zn^II^ ion has a distorted trigonal-bipyramidal coordination environment. In the crystal, there exist π-π stacking interactions involving symmetry related 2,6-dipyrazol-1-ylpyridine ligands (Talbe 1).

### Experimental

15 ml me thanol solution containing 2,6-dipyrazol-1-ylpyridine (0.0522 g, 0.247 mmol) and pyrazine-1,4-dioxide (0.0414 g, 0.369 mmol) was added into 5 ml H_2_O solution of ZnCl_2_ (0.0783 g, 0.575 mmol), and the mixed solution was stirred for a few minutes. Colorless single crystals were obtained after the filtrate was allowed to stand at room temperature for 40 days.

### Refinement

All H atoms were placed in calculated positions with C—H = 0.93 Å and refined as riding with *U*_iso_(H) = 1.2*U*_eq_(C).

### Crystal data

**Table:**

[ZnCl_2_(C_11_H_9_N_5_)]	*F*(000) = 696
*M**_r_* = 347.50	*D*_x_ = 1.758 Mg m^−^^3^
Monoclinic, *P*2_1_/*c*	Mo *K*α radiation, λ = 0.71073 Å
Hall symbol: -P 2ybc	Cell parameters from 3889 reflections
*a* = 10.9630 (17) Å	θ = 2.4–28.2°
*b* = 8.0263 (13) Å	µ = 2.27 mm^−^^1^
*c* = 14.943 (2) Å	*T* = 298 K
β = 93.079 (2)°	Block, colourless
*V* = 1313.0 (4) Å^3^	0.48 × 0.42 × 0.29 mm
*Z* = 4	

### Data collection

**Table:**

Bruker SMART APEX CCD diffractometer	2848 independent reflections
Radiation source: fine-focus sealed tube	2431 reflections with *I* > 2σ(*I*)
graphite	*R*_int_ = 0.030
φ and ω scans	θ_max_ = 27.0°, θ_min_ = 2.9°
Absorption correction: multi-scan (SADABS; Sheldrick, 1996)	*h* = −9→14
*T*_min_ = 0.409, *T*_max_ = 0.559	*k* = −9→10
7375 measured reflections	*l* = −16→18

### Refinement

**Table:**

Refinement on *F*^2^	Secondary atom site location: difference Fourier map
Least-squares matrix: full	Hydrogen site location: inferred from neighbouring sites
*R*[*F*^2^ > 2σ(*F*^2^)] = 0.029	H-atom parameters constrained
*wR*(*F*^2^) = 0.078	*w* = 1/[σ^2^(*F*_o_^2^) + (0.0445*P*)^2^ + 0.0869*P*] where *P* = (*F*_o_^2^ + 2*F*_c_^2^)/3
*S* = 1.05	(Δ/σ)_max_ = 0.002
2848 reflections	Δρ_max_ = 0.28 e Å^−^^3^
173 parameters	Δρ_min_ = −0.32 e Å^−^^3^
0 restraints	Extinction correction: SHELXTL (Sheldrick, 2008), Fc^*^=kFc[1+0.001xFc^2^λ^3^/sin(2θ)]^-1/4^
Primary atom site location: structure-invariant direct methods	Extinction coefficient: 0.0167 (11)

### Special details

**Table:**

Geometry. All e.s.d.'s (except the e.s.d. in the dihedral angle between two l.s. planes) are estimated using the full covariance matrix. The cell e.s.d.'s are taken into account individually in the estimation of e.s.d.'s in distances, angles and torsion angles; correlations between e.s.d.'s in cell parameters are only used when they are defined by crystal symmetry. An approximate (isotropic) treatment of cell e.s.d.'s is used for estimating e.s.d.'s involving l.s. planes.
Refinement. Refinement of *F*^2^ against ALL reflections. The weighted *R*-factor *wR* and goodness of fit *S* are based on *F*^2^, conventional *R*-factors *R* are based on *F*, with *F* set to zero for negative *F*^2^. The threshold expression of *F*^2^ > σ(*F*^2^) is used only for calculating *R*-factors(gt) *etc*. and is not relevant to the choice of reflections for refinement. *R*-factors based on *F*^2^ are statistically about twice as large as those based on *F*, and *R*- factors based on ALL data will be even larger.

### Fractional atomic coordinates and isotropic or equivalent isotropic displacement parameters (Å^2^)

**Table:**

	*x*	*y*	*z*	*U*_iso_*/*U*_eq_	
C1	0.47097 (18)	0.3058 (2)	0.04770 (16)	0.0447 (5)	
H1	0.4465	0.3525	0.1008	0.054*	
C2	0.40961 (19)	0.3275 (3)	−0.03549 (17)	0.0478 (5)	
H2	0.3389	0.3891	−0.0481	0.057*	
C3	0.47479 (18)	0.2396 (2)	−0.09475 (16)	0.0440 (5)	
H3	0.4572	0.2292	−0.1561	0.053*	
C4	0.66172 (17)	0.0609 (2)	−0.07399 (13)	0.0349 (4)	
C5	0.66885 (19)	0.0026 (3)	−0.16015 (14)	0.0448 (5)	
H5	0.6147	0.0387	−0.2062	0.054*	
C6	0.7591 (2)	−0.1111 (3)	−0.17553 (16)	0.0487 (5)	
H6	0.7663	−0.1533	−0.2330	0.058*	
C7	0.8394 (2)	−0.1637 (3)	−0.10659 (15)	0.0442 (5)	
H7	0.8998	−0.2423	−0.1160	0.053*	
C8	0.82578 (17)	−0.0944 (2)	−0.02369 (14)	0.0357 (4)	
C9	0.99689 (18)	−0.2410 (2)	0.06325 (16)	0.0454 (5)	
H9	1.0275	−0.3088	0.0192	0.054*	
C10	1.0387 (2)	−0.2314 (3)	0.14943 (17)	0.0502 (6)	
H10	1.1036	−0.2903	0.1767	0.060*	
C11	0.9647 (2)	−0.1152 (3)	0.18931 (17)	0.0491 (6)	
H11	0.9730	−0.0837	0.2492	0.059*	
Cl1	0.65199 (5)	0.06053 (7)	0.25184 (4)	0.05045 (17)	
Cl2	0.83525 (5)	0.37392 (6)	0.12155 (4)	0.04545 (15)	
N1	0.74008 (13)	0.01621 (18)	−0.00710 (10)	0.0327 (3)	
N2	0.90163 (15)	−0.13336 (19)	0.05243 (12)	0.0374 (4)	
N3	0.88095 (15)	−0.0554 (2)	0.13084 (11)	0.0417 (4)	
N4	0.57069 (14)	0.1699 (2)	−0.04655 (11)	0.0366 (4)	
N5	0.56823 (14)	0.2105 (2)	0.04169 (11)	0.0385 (4)	
Zn1	0.73202 (2)	0.13073 (3)	0.121755 (15)	0.03696 (11)	

### Atomic displacement parameters (Å^2^)

**Table:**

	*U*^11^	*U*^22^	*U*^33^	*U*^12^	*U*^13^	*U*^23^
C1	0.0377 (11)	0.0347 (10)	0.0624 (14)	−0.0040 (8)	0.0090 (10)	−0.0009 (10)
C2	0.0347 (10)	0.0363 (10)	0.0721 (16)	−0.0005 (9)	−0.0004 (10)	0.0102 (10)
C3	0.0360 (10)	0.0439 (11)	0.0512 (13)	−0.0045 (9)	−0.0066 (9)	0.0124 (9)
C4	0.0329 (10)	0.0332 (9)	0.0386 (11)	−0.0072 (7)	0.0014 (8)	0.0020 (8)
C5	0.0441 (11)	0.0538 (12)	0.0361 (11)	−0.0052 (10)	−0.0025 (9)	0.0023 (9)
C6	0.0539 (13)	0.0570 (13)	0.0357 (12)	−0.0052 (10)	0.0062 (10)	−0.0076 (10)
C7	0.0428 (12)	0.0470 (11)	0.0435 (12)	0.0032 (9)	0.0071 (9)	−0.0029 (9)
C8	0.0338 (10)	0.0337 (9)	0.0400 (11)	−0.0062 (8)	0.0054 (8)	0.0026 (8)
C9	0.0392 (11)	0.0374 (11)	0.0599 (15)	0.0031 (9)	0.0064 (10)	0.0053 (9)
C10	0.0414 (11)	0.0469 (12)	0.0613 (15)	0.0030 (9)	−0.0047 (10)	0.0154 (11)
C11	0.0458 (12)	0.0549 (13)	0.0460 (14)	0.0010 (10)	−0.0036 (10)	0.0090 (10)
Cl1	0.0523 (3)	0.0611 (4)	0.0389 (3)	−0.0069 (2)	0.0112 (2)	−0.0012 (2)
Cl2	0.0431 (3)	0.0428 (3)	0.0500 (3)	−0.0086 (2)	−0.0017 (2)	−0.0035 (2)
N1	0.0309 (8)	0.0332 (8)	0.0338 (9)	−0.0042 (6)	0.0000 (7)	0.0014 (7)
N2	0.0348 (9)	0.0376 (9)	0.0400 (10)	0.0006 (6)	0.0034 (7)	0.0025 (7)
N3	0.0413 (9)	0.0486 (10)	0.0351 (9)	0.0030 (8)	0.0022 (7)	0.0011 (7)
N4	0.0316 (8)	0.0370 (8)	0.0407 (10)	−0.0038 (7)	−0.0015 (7)	0.0037 (7)
N5	0.0359 (9)	0.0376 (9)	0.0419 (10)	−0.0029 (7)	0.0022 (7)	−0.0007 (7)
Zn1	0.03632 (16)	0.04045 (17)	0.03418 (17)	−0.00377 (9)	0.00263 (10)	−0.00260 (9)

### Geometric parameters (Å, °)

**Table:**

C1—N5	1.319 (3)	C8—N1	1.325 (2)
C1—C2	1.392 (3)	C8—N2	1.408 (3)
C1—H1	0.9300	C9—C10	1.346 (3)
C2—C3	1.364 (3)	C9—N2	1.358 (2)
C2—H2	0.9300	C9—H9	0.9300
C3—N4	1.362 (2)	C10—C11	1.391 (3)
C3—H3	0.9300	C10—H10	0.9300
C4—N1	1.332 (2)	C11—N3	1.323 (3)
C4—C5	1.376 (3)	C11—H11	0.9300
C4—N4	1.405 (2)	Cl1—Zn1	2.2470 (6)
C5—C6	1.374 (3)	Cl2—Zn1	2.2564 (6)
C5—H5	0.9300	N1—Zn1	2.1397 (16)
C6—C7	1.385 (3)	N2—N3	1.358 (2)
C6—H6	0.9300	N3—Zn1	2.2117 (17)
C7—C8	1.374 (3)	N4—N5	1.360 (2)
C7—H7	0.9300	N5—Zn1	2.1988 (16)
			
Cg1···Cg2^i^	3.4087 (12)	Cg2···Cg3^i^	3.6253 (13)
			
N5—C1—C2	111.4 (2)	C11—C10—H10	127.1
N5—C1—H1	124.3	N3—C11—C10	111.1 (2)
C2—C1—H1	124.3	N3—C11—H11	124.4
C3—C2—C1	105.67 (19)	C10—C11—H11	124.4
C3—C2—H2	127.2	C8—N1—C4	118.37 (17)
C1—C2—H2	127.2	C8—N1—Zn1	121.33 (13)
N4—C3—C2	106.6 (2)	C4—N1—Zn1	120.23 (13)
N4—C3—H3	126.7	C9—N2—N3	110.69 (17)
C2—C3—H3	126.7	C9—N2—C8	130.90 (18)
N1—C4—C5	122.93 (18)	N3—N2—C8	118.41 (16)
N1—C4—N4	112.86 (17)	C11—N3—N2	105.09 (18)
C5—C4—N4	124.19 (18)	C11—N3—Zn1	140.30 (16)
C6—C5—C4	117.4 (2)	N2—N3—Zn1	114.53 (12)
C6—C5—H5	121.3	N5—N4—C3	111.07 (17)
C4—C5—H5	121.3	N5—N4—C4	118.92 (16)
C5—C6—C7	120.8 (2)	C3—N4—C4	129.90 (18)
C5—C6—H6	119.6	C1—N5—N4	105.25 (17)
C7—C6—H6	119.6	C1—N5—Zn1	140.46 (15)
C8—C7—C6	116.84 (19)	N4—N5—Zn1	113.56 (11)
C8—C7—H7	121.6	N1—Zn1—N5	72.99 (6)
C6—C7—H7	121.6	N1—Zn1—N3	72.49 (6)
N1—C8—C7	123.55 (19)	N5—Zn1—N3	143.97 (6)
N1—C8—N2	113.06 (17)	N1—Zn1—Cl1	135.05 (4)
C7—C8—N2	123.38 (18)	N5—Zn1—Cl1	101.44 (5)
C10—C9—N2	107.3 (2)	N3—Zn1—Cl1	95.67 (5)
C10—C9—H9	126.3	N1—Zn1—Cl2	108.99 (4)
N2—C9—H9	126.3	N5—Zn1—Cl2	98.18 (5)
C9—C10—C11	105.8 (2)	N3—Zn1—Cl2	102.45 (5)
C9—C10—H10	127.1	Cl1—Zn1—Cl2	115.93 (2)
			
N5—C1—C2—C3	−0.1 (2)	C5—C4—N4—N5	175.69 (18)
C1—C2—C3—N4	−0.1 (2)	N1—C4—N4—C3	−178.85 (18)
N1—C4—C5—C6	2.3 (3)	C5—C4—N4—C3	−0.2 (3)
N4—C4—C5—C6	−176.27 (18)	C2—C1—N5—N4	0.2 (2)
C4—C5—C6—C7	−0.2 (3)	C2—C1—N5—Zn1	169.07 (15)
C5—C6—C7—C8	−1.3 (3)	C3—N4—N5—C1	−0.3 (2)
C6—C7—C8—N1	0.9 (3)	C4—N4—N5—C1	−176.86 (16)
C6—C7—C8—N2	−178.97 (18)	C3—N4—N5—Zn1	−172.54 (12)
N2—C9—C10—C11	0.3 (2)	C4—N4—N5—Zn1	10.85 (19)
C9—C10—C11—N3	0.0 (3)	C8—N1—Zn1—N5	−173.52 (14)
C7—C8—N1—C4	1.0 (3)	C4—N1—Zn1—N5	9.64 (13)
N2—C8—N1—C4	−179.07 (15)	C8—N1—Zn1—N3	−4.01 (13)
C7—C8—N1—Zn1	−175.88 (15)	C4—N1—Zn1—N3	179.15 (15)
N2—C8—N1—Zn1	4.0 (2)	C8—N1—Zn1—Cl1	−84.26 (14)
C5—C4—N1—C8	−2.7 (3)	C4—N1—Zn1—Cl1	98.90 (13)
N4—C4—N1—C8	176.00 (15)	C8—N1—Zn1—Cl2	93.49 (13)
C5—C4—N1—Zn1	174.24 (14)	C4—N1—Zn1—Cl2	−83.35 (13)
N4—C4—N1—Zn1	−7.1 (2)	C1—N5—Zn1—N1	−178.6 (2)
C10—C9—N2—N3	−0.4 (2)	N4—N5—Zn1—N1	−10.30 (11)
C10—C9—N2—C8	179.88 (19)	C1—N5—Zn1—N3	164.27 (19)
N1—C8—N2—C9	178.77 (18)	N4—N5—Zn1—N3	−27.46 (18)
C7—C8—N2—C9	−1.3 (3)	C1—N5—Zn1—Cl1	47.6 (2)
N1—C8—N2—N3	−0.9 (2)	N4—N5—Zn1—Cl1	−144.18 (11)
C7—C8—N2—N3	179.04 (18)	C1—N5—Zn1—Cl2	−71.1 (2)
C10—C11—N3—N2	−0.3 (2)	N4—N5—Zn1—Cl2	97.15 (12)
C10—C11—N3—Zn1	−176.68 (16)	C11—N3—Zn1—N1	179.3 (2)
C9—N2—N3—C11	0.4 (2)	N2—N3—Zn1—N1	3.17 (12)
C8—N2—N3—C11	−179.84 (17)	C11—N3—Zn1—N5	−163.4 (2)
C9—N2—N3—Zn1	177.92 (12)	N2—N3—Zn1—N5	20.38 (19)
C8—N2—N3—Zn1	−2.4 (2)	C11—N3—Zn1—Cl1	−45.1 (2)
C2—C3—N4—N5	0.2 (2)	N2—N3—Zn1—Cl1	138.76 (12)
C2—C3—N4—C4	176.33 (18)	C11—N3—Zn1—Cl2	73.1 (2)
N1—C4—N4—N5	−3.0 (2)	N2—N3—Zn1—Cl2	−103.08 (12)

Symmetry codes: (i) −*x*+1, −*y*, −*z*.
